# Report of a Rare Case of Gorham-Stout Disease of Both Shoulders: Bisphosphonate Treatment and Shoulder Replacement

**DOI:** 10.1155/2011/565142

**Published:** 2011-10-19

**Authors:** Eike Garbers, Falk Reuther, Gunther Delling

**Affiliations:** ^1^Klinik für Unfallchirurgie und Orthopaedie, DRK Kliniken Berlin Koepenick, Salvador-Allende-Straße 2-8, 12559 Berlin, Germany; ^2^Institut für Pathologie Hannover, Berliner Allee 48, 30175 Hannover, Germany

## Abstract

Massive osteolysis known as Gorham-Stout disease is a rare idiopathic disorder typically affecting long bones in a unifocal pattern. Angiomatosis is strongly connected to the osteolysis. Weather angiomatosis is the cause or the result of osteolysis is subject of intense discussion (Kawasaki et al. (2003), Möller et al. (1999), Radhakrishnan and Rockson (2008)). There are about 200 cases described since 1955. Our patient is a 77-year-old female patient with osteolyses of both shoulders involving the proximal humerus, lateral clavicle, and the glenoid. Under bisphosphonate therapy, the progressive osteolysis stopped on the right side and showed progression on the left. With the patient complaining about severe rest pain and impaired function, we performed surgical reconstruction by implantation of total shoulder prosthesis three months after onset of symptoms. Our case shows a possibility of primary and early surgical reconstruction with good clinical outcome.

## 1. Introduction

Osteolyses are common findings on plain X-rays of the skeletal system. Most commonly, they are seen as secondary osteolyses due to an underlying hereditary, metabolic, or neoplastic condition [1, 2]. From these secondary osteolyses, we differentiate the rare group of idiopathic primary osteolyses. Hardegger et al. classified these in five groups as shown in Table 1 [1]. 

The Gorham-Stout disease (GSD; synonyms: massive osteolysis, vanishing bone disease) is a rare condition of unknown etiology characterized by semispecific histological findings and a typical radiological and clinical pattern resulting in progressive destruction and resorption of bony structures [3]. Typical histological findings include local osteoclastic hyperactivity and proliferation of small thin-walled arterial or lymphatic vessels (angiomatosis) [3–7]; radiological features include massive progressive osteolysis that does not respect anatomical borders and spread from the primary focus to adjoining bones [2, 8]. The idiopathic osteolysis is self-limiting after a variable amount of time. There is no specific treatment described yet; symptomatic therapy includes bisphosphonate medication, alpha-2b-interferon, radiation therapy, and surgical procedures as resection and endoprosthetic operation [9–12].

GSD was defined first by Gorham and Stout in 1955, where the authors suspected the local angiomatosis to result in a local hyperemia resulting in an alteration of the balance of osteoblastic and osteoclastic activity thus leading to massive osteolysis. In many cases, a minor trauma was described as an initiating event for the osteolysis and the authors hypothesized that the proliferation of a local angiomatosis might be triggered by a trauma mechanism [3].

The age of onset is variable; in about 200 reported cases, the majority were younger patients; there were no familiar disposition and no gender specific distribution. Typical unifocal localizations are the skull (18.3%), the pelvis (17.7%), the shoulder girdle (16.0%), the lower (14.9%), and the upper limb (11.4%); less often the spine, ribs, and the sternum were reported to be affected. The overall lethality is about 13.3%; involvement of the thorax typically including a chylothorax result in a poor prognosis with a lethality of 52% [13–15]. Diagnostic criteria for the GSD are given in Table 2 and include radiological, histological, and clinical features after ruling out of hereditary, metabolic, neoplastic, infectious, or immunological sources of osteolyses [4, 13, 16]. 

## 2. Case Description

**Figure 1:**
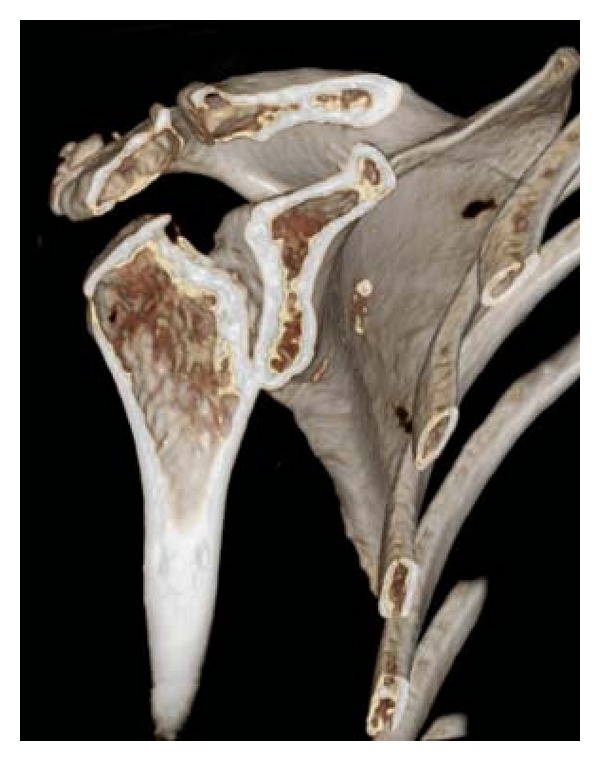
3D reconstructed CT scan showing osteonecrosis of the humeral head, the acromion, lateral clavicle, and the glenoid in Gorham-Stout disease.

**Figure 2:**
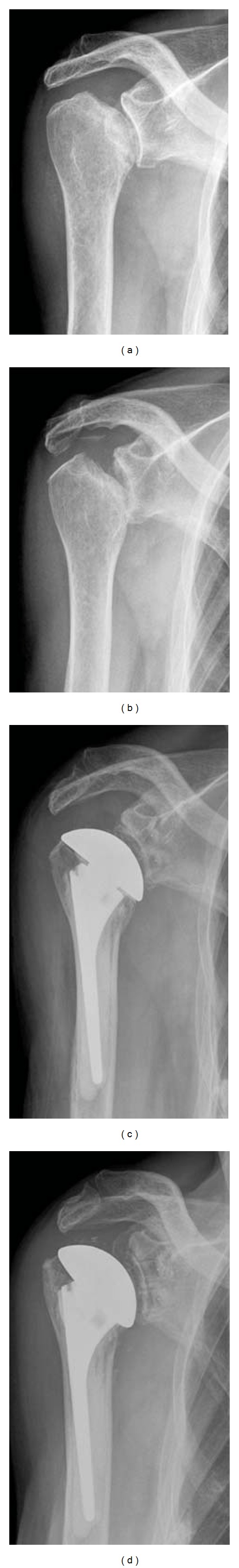
Plain true a.p. X-rays of the right shoulder on first admission in 12/2007 (a), after disease limitation under bisphosphonate therapy in 03/2008 (b), postoperative after implantation of total shoulder prosthesis (c), 1-year followup showing no progression of the osteolyses and no signs of loosening of the prosthesis (d).

**Figure 3:**
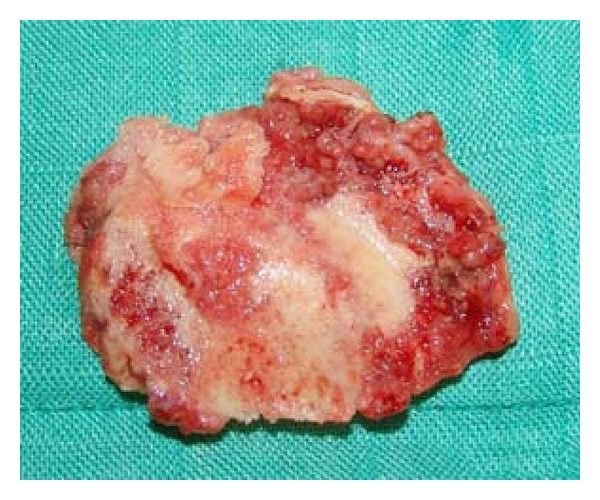
Macroscopic specimen of the humeral head showing massive osteolysis.

**Figure 4:**
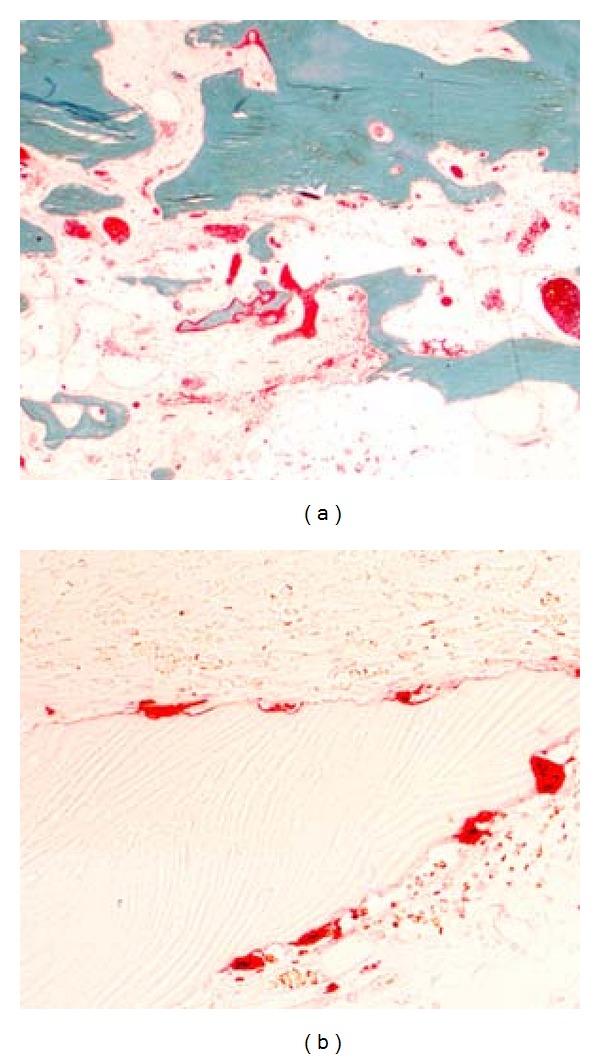
Histological stains showing altered osteoclastic resorption (acid phosphatase reaction 200x) (a); cortical and cancellous bone with stimulated turnover and marrow fibrosis (Goldner) (b).

A 77-year-old woman who complained accentuated rest pain in both shoulders 8 weeks after minor trauma was admitted to our clinic. Past medical history includes chronic bronchial asthma without steroid medication, hyperthyroidism (radiotherapy 2005), cerebrovascular event without residual neurologic deficiency (1998), and osteoporosis with three osteoporotic lumbar spine fractures (vertebroplasty 2006). Plain X-rays of both shoulders showed osteolyses of both humeral heads, glenoids, and lateral clavicles (Figures 1 and 2(a)). After ruling out of secondary osteolysis, we started the patient on bisphosphonates (alendronate 70 mg per week), calcium, and calcitonin. Early follow-up X-rays showed the termination of progressive osteolyses (Figure 2(b)). After clinical and radiological termination of the progressive disease after three months, the patient was readmitted with poor function and constant rest pain accentuated on the right side. After obtaining CT and MRI scans, we performed reconstruction on the right side by implantation of anatomical shoulder prosthesis (AFFINIS, Mathys Ltd., Switzerland). Intraoperative findings where massive synovitis with significant thickening of the capsule and massive osteolysis of the humeral head (Figure 3) and the glenoid. The rotator cuff was not affected. Pathological and microbiological specimens where taken and sent to a specialized laboratory. The cemented prosthesis was implanted in typical fashion; the glenoid was resurfaced with a polyethylene component. Microbiological specimens came back negative. Pathological findings confirmed macroscopic findings of a detritus-synovitis, massive osteoclastic resorption with stimulated osteoclastic resorption, edema, and fibroses of the bone marrow. These findings were consistent with Gorham-Stout syndrome (Figure 4). 

On postoperative followup 1 year after surgery, the patient presented with significant improvement in function and pain. The ASES-Score [17] improved from 9.6 to 35, constant score [18] improved from 11% to 34%, and DASH score [14] improved from 81.6 to 59. Plain X-rays showed a correctly implanted prosthesis without signs of loosening; in the glenoid and the lateral clavicle we found minor progression of the osteolysis (Figure 2(d)).

## 3. Discussion

**Figure 5:**
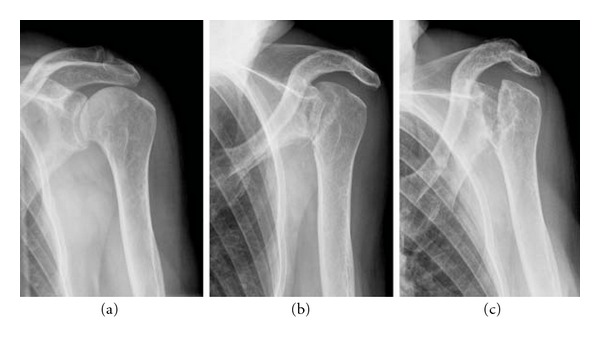
Plain true a.p. X-rays of the left shoulder in the same patient showing progressive osteolyses of the humeral head, the acromion, the lateral clavicle, and the glenoid in Gorham-Stout disease.

Our case is the first reported case of bilateral shoulder involvement in GSD. We were able to show the benefit of bisphosphonate therapy in disease control of Gorham Stout as the progression stopped about 3 month after initiation. The effectiveness of this therapy was described in detail before, and we can fully confirm these results [9, 12, 19]. Eventually, the osteolysis stopped in an early stage with limited bone loss. Because the patient complained of limited function and severe pain in both shoulders, we decided to do perform prosthetic replacement of the right glenohumeral joint. The left side showed comparable radiologic findings but the patient did not complain of severe pain and did not require surgical therapy (Figure 5). One year after surgery, the patient reported significant improvement in function and pain relief in the right shoulder represented in almost threefold enhancement in the shoulder scores as shown in results. For the timing of surgery, there is no clear guideline and surgeons are concerned about progression of the osteolysis resulting in loosening of the prosthesis. We performed a relatively early operation and achieved a good result without loosening of the prosthesis [20]. The histopathological specimens showed significant osteoclastic resorption with fibrosis and edema of the bone marrow. Even though the histological findings were not characteristic for the Gorham-Stout disease as they were described in the original paper, we made the diagnosis from the massive osteoclastic resorption without osteoblastic stimulation together with the classical clinical and radiological findings [1, 6, 15]. Histological specimens showed no ulcers; secondary osteonecrosis was ruled out by CT of the abdomen, laboratory tests, chest X-ray, and colonoscopy. Kawasaki et al. reported on an autopsy case of GSD and discussed our question—if angiomatosis is an intrinsic pathohistological feature of GSD—in detail [5]. They did not find any of the vascular changes in cases of GSD consistent with other authors before. They concluded that vascular proliferation might not always be associated with GSD but may be one of the results of disease rather than the cause [5].

## Figures and Tables

**Table 1:** Classification of idiopathic osteolyses [1].

Type 1: Hereditary multifocal osteolyses with dominant inheritance	
Type 2: Hereditary multifocal osteolyses with recessive inheritance	
Type 3: Nonhereditary multifocal osteolyses with nephropathia	
Type 4: Gorham-Stout syndrome	
Type 5: Winchester syndrome	

**Table 2:** Criteria for the diagnosis of Gorham-Stout-disease [13].

(i) Positive histology (angiomatosis)	
(ii) Absence of cellular atypicalities	
(iii) Minimal or no osteoplastic reaction	
(iv) Local progressing resorption of bone	
(v) Absence of ulcerations	
(vi) Absence of visceral involvement	
(vii) Radiological finding of osteolysis	
(viii) Ruling out of hereditary, metabolic, neoplastic, infectious, or immunological etiology	

## References

[1] Hardegger F, Simpson LA, Segmueller G (1985). The syndrome of idiopathic osteolysis. Classification, review, and case report. *Journal of Bone and Joint Surgery*.

[2] Patel DV (2005). Gorham's disease or massive osteolysis. *Clinical Medicine & Research*.

[3] Gorham LW, Stout AP (1955). Massive osteolysis (acute spontaneous absorption of bone, phantom bone, disappearing bone); its relation to hemangiomatosis. *The Journal of Bone and Joint Surgery*.

[4] Bruch-Gerharz D, Gerharz CD, Stege H (2003). Cutaneous Vascular Malformations in Disappearing Bone (Gorham-Stout) Disease. *JAMA*.

[5] Kawasaki K, Ito T, Tsuchiya T, Takahashi H (2003). Is angiomatosis an intrinsic pathohistological feature of massive osteolysis? Report of an autopsy case and a review of the literature. *Virchows Archiv*.

[6] Möller G, Gruber H, Priemel M, Werner M, Kuhlmey AS, Delling G (1999). Gorham-Stout idiopathic osteolysis—a local osteoclastic hyperactivity?. *Pathologe*.

[7] Radhakrishnan K, Rockson SG (2008). Gorham's disease: an osseous disease of lymphangiogenesis?. *Annals of the New York Academy of Sciences*.

[8] Boyer P, Bourgeois P, Boyer O, Catonné Y, Saillant G (2005). Massive Gorham-Stout syndrome of the pelvis. *Clinical Rheumatology*.

[9] Lehmann G, Pfeil A, Böttcher J (2009). Benefit of a 17-year long-term bisphosphonate therapy in a patient with Gorham-Stout syndrome. *Archives of Orthopaedic and Trauma Surgery*.

[10] Pfleger A, Schwinger W, Maier A, Tauss J, Popper HH, Zach MS (2006). Gorham-Stout syndrome in a male adolescent—case report and review of the literature. *Journal of Pediatric Hematology/Oncology*.

[11] Takahashi A, Ogawa C, Kanazawa T (2005). Remission induced by interferon alfa in a patient with massive osteolysis and extension of lymph-hemangiomatosis: a severe case of Gorham-Stout syndrome. *Journal of Pediatric Surgery*.

[12] Hammer F, Kenn W, Wesselmann U (2005). Gorham-Stout disease—stabilization during bisphosphonate treatment. *Journal of Bone and Mineral Research*.

[13] Florchinger A, Bottger E, Claass-Bottger F, Georgi M, Harms J (1998). Gorham-Stout syndrome of the spine. Case report and review of the literature. *Rofo*.

[14] Germann G, Wind G, Harth A (1999). The DASH(Disability of Arm-Shoulder-Hand) Questionnaire—a new instrument for evaluating upper extremity treatment outcome. *Handchir Mikrochir Plast Chir*.

[15] Moller G, Priemel M, Amling M, Werner M, Kuhlmey AS, Delling G (1999). The Gorham-Stout syndrome (Gorham's massive osteolysis). A report of six cases with histopathological findings. *Journal of Bone and Joint Surgery*.

[16] Bruch-Gerharz D, Gerharz CD, Stege H (2007). Cutaneous lymphatic malformations in disappearing bone (Gorham-Stout) disease: a novel clue to the pathogenesis of a rare syndrome. *Journal of the American Academy of Dermatology*.

[17] McClure PM, Michener L (2003). Measures of adult shoulder function. *Arthritis & Rheumatism*.

[18] Constant CR, Murley AH (1987). A clinical method of functional assessment of the shoulder. *Clinical Orthopaedics and Related Research*.

[19] Lehnhardt M, Steinau HU, Homann HH, Steinstraesser L, Druecke D (2004). Gorham-Stout disease: report of a case affecting the right hand with a follow-up of 24 years. *Handchirurgie Mikrochirurgie Plastische Chirurgie*.

[20] Sestan B, Miletic D (2006). Rapid idiopathic osteolysis of the humeral head and clavicle. *West Indian Medical Journal*.

